# Multiple metrics assessment method for a reliable evaluation of corneal suturing skills

**DOI:** 10.1038/s41598-023-29555-3

**Published:** 2023-02-20

**Authors:** Lea Dormegny, Nicole Neumann, Anne Lejay, Arnaud Sauer, David Gaucher, François Proust, Nabil Chakfe, Tristan Bourcier

**Affiliations:** 1 GEPROMED, Strasbourg, France; 2 Department of Ophthalmology, Nouvel Hôpital Civil, Strasbourg University Hospital, BP426, 67091 Strasbourg Cedex, France; 3 UNISIMES (UNIté de SIMulation Européenne en Santé), Strasbourg University Hospital, Faculty of Medicine, Strasbourg, France; 4 Department of Vascular Surgery and Kidney Transplantation, Strasbourg University Hospital, Strasbourg, France; 5 Department of Neurosurgery, Strasbourg University Hospital, Strasbourg, France

**Keywords:** Outcomes research, Clinical trial design

## Abstract

This study aimed to evaluate the efficiency of a multiple metrics assessment method to differentiate between surgeons of differing experience while performing a corneal suturing task. Volunteer ophthalmologists were assigned to three groups (senior [SG], junior [JG] and novice [NG]) according to their experience in corneal suturing. All participants performed three sessions of corneal wound closure by three stitches. Suturing and participant posture were recorded with cameras, and assessed by two blind assessors for stitch quality (using Zhang score) and ergonomics (using Rapid Upper Limb Assessment [RULA] score). Task duration was recorded. Objective analyses of stitches geometry and instrument position were carried out. We included 24 participants: 5 in the SG, 8 in the JG and 11 in the NG. Stitch quality was significantly better and time to perform the procedure significantly lower in more experienced groups (p < 0.001 and p = 0.002, respectively). SG participants better respected regular distance and parallelism between stitches compared to others (p = 0.01). Instrument position was similar between groups, although SG participants minimized their back-and-forth movements compared to NG participants. Ergonomics assessment was similar. Multiple metrics assessment efficiently determined how to differentiate between novices and experienced surgeons on corneal suturing skills, providing hints for future training studies.

## Introduction

The performance and assessment of surgical procedures on simulated tissues has been promoted by the emergence of surgical training programs^1^. Such programs circumvent the need for novices to begin their practice on real patients. However, actual training programs greatly diverge with regard to both the parameters and methods of assessment^2,3^. Careful selection of these latter is of major importance for the reproducibility and reliability of surgical skills assessments^4^.

Corneal suturing is a fundamental skill in ophthalmic surgery, which should be mastered in the early stages of residency. Therefore, anticipatory training for its practice should be promoted to improve surgical outcome and reduce the risk of complications in many ophthalmic procedures^5^. Whilst few studies report dedicated training programs^3^, assessment tools for corneal suturing remain underdeveloped^6,7^.

Comparing the performance of the same surgical procedure by both novice and experienced surgeons would allow to objectively identify parameters where stark differences occur according to surgical experience. These parameters could provide trainees with specific metrics to assess their skill level and to focus on during training.

This study aimed to evaluate the utility of an assessment method for corneal suturing skills combining multiple metrics to assess ergonomics, and suturing quality and rapidity. To do so, we prospectively compared the performance of a corneal wound closure task for three groups of ophthalmic surgeons of varying experience—novice, junior, and senior.

## Results

We enrolled 24 participants in the study. Five (20.8%) were placed in the SG (senior group), 8 (33.3%) in the JG (junior group) and 11 (45.8%) in the NG (novice group). Of the participants in the SG, two surgeons routinely performed corneal stitches, two others were pediatric ophthalmic surgeons routinely performing strabismus and cataract surgery and one was a vitreoretinal surgeon. Participants from the JG did not have regular surgical activity and performed corneal stitches intermittently. The wound in each transplant was considered identical upon inspection of the recordings. All participants completed the three suturing sessions. Several participants from the SG and the JG carried out a significant number of corneal stitches between sessions (mean of 22.0 ± 31.2 stitches for the SG and 3.9 ± 6.5 for the JG), while participants from the NG did not perform any. Considering this, the results of the three sessions were pooled together for statistical analysis in the NG, whilst only the first session was considered for the JG and the SG. The gain of experience from one session to another was considered negligible in the NG due to the short format of the suturing exercise and the fairly long time interval between two sessions, while participants from the JG and SG carried out many stitches between two sessions, which might have influenced their results.

### Achievement

All SG and JG participants managed to achieve the three stitches required in each session. Among the NG participants, 21.1% (N = 7) were not able to finish a single stitch across all sessions, whilst 72.7% (N = 24) achieved all three stitches (Table 1).

**Table 1:** Characteristics of the performance of Senior, Junior and Novice groups during a corneal wound closure task, according to type of analysis. *IQR* interquartile range, *mm* millimeters, *RULA* Rapid Upper Limb Assessment. Significant values are in bold.

	Senior group	Junior group	Novice group	p value
Number of participants	5	8	33	–
Number of corneal stitches previously carried out (mean)	> 100	23.5	1.64	–
Achieved stitches (% participants)				
None	0 (0)	0 (0)	7 (21.2)	–
1	0 (0)	0 (0)	1 (3.03)	–
2	0 (0)	0 (0)	1 (3.03)	–
3	5 (100)	8 (100)	24 (72.7)	–
Subjective assessments, median (IQR)				
Zhang score (/60)	58.0 (58.0–59.0)	50.5 (47.0–54.5)	36.0 (28.0–43.0)	0.0000106*
RULA score (/10)	3.00 (3.00–3.00)	3.00 (3.00–5.25)	3.00 (3.00–6.00)	0.303
Time analysis (min), median (IQR)				
Total time with penalties	6.90 (5.77–7.23)	7.97 (6.75–9.84)	13.1 (10.6–18.9)	0.00185*
Total time without penalties	6.90 (5.77–7.23)	7.97 (6.75–9.84)	12.2 (7.92–13.2)	0.0175*
Stitch 1	2.20 (1.52–2.47)	3.07 (1.83–3.58)	4.18 (2.82–6.12)	0.0128*
Stitch 2	1.70 (1.12–1.83)	1.85 (1.30–2.33)	2.25 (1.85–3.43)	0.0317*
Stitch 3	1.50 (1.37–1.68)	1.78 (1.51–2.20)	2.75 (1.46–3.73)	0.158
Intermediate time	1.12 (1.08–1.52)	0.95 (0.90–1.16)	1.30 (1.06–2.95)	0.122
Zone analysis (% time in zone), median (IQR)				
Zone 1	73.6 (62.4–77.5)	51.6 (50.9–62.3)	60.6 (47.1–74.3)	0.152
Zone 2	31.8 (24.0–32.8)	37.1 (33.8–39.0)	36.3 (24.6–44.1)	0.681
Zone 3	21.0 (20.3–28.8)	23.8 (16.1–27.6)	21.0 (16.7–27.7)	0.943
Geometry analysis, median (IQR)				
Length of each stitch (mm)	1.09 (0.89–1.37)	0.85 (0.749–1.38)	1.15 (0.94–1.47)	0.0750
Length ratio with respect to stitch 1	1.16 (1.03–1.28)	1.05 (0.87–1.24)	0.83 (0.67–1.02)	0.0114*
Distance between successive stitches (mm)	0.94 (0.81–1.15)	0.78 (0.62–0.953)	0.88 (0.74–1.02)	0.241
Angle between successive stitches (°)	2.80 (1.73–6.30)	5.75 (2.75–6.82)	6.86 (3.55–12.2)	0.0690

### Performance assessment by Zhang and RULA scores

**Figure 1:**
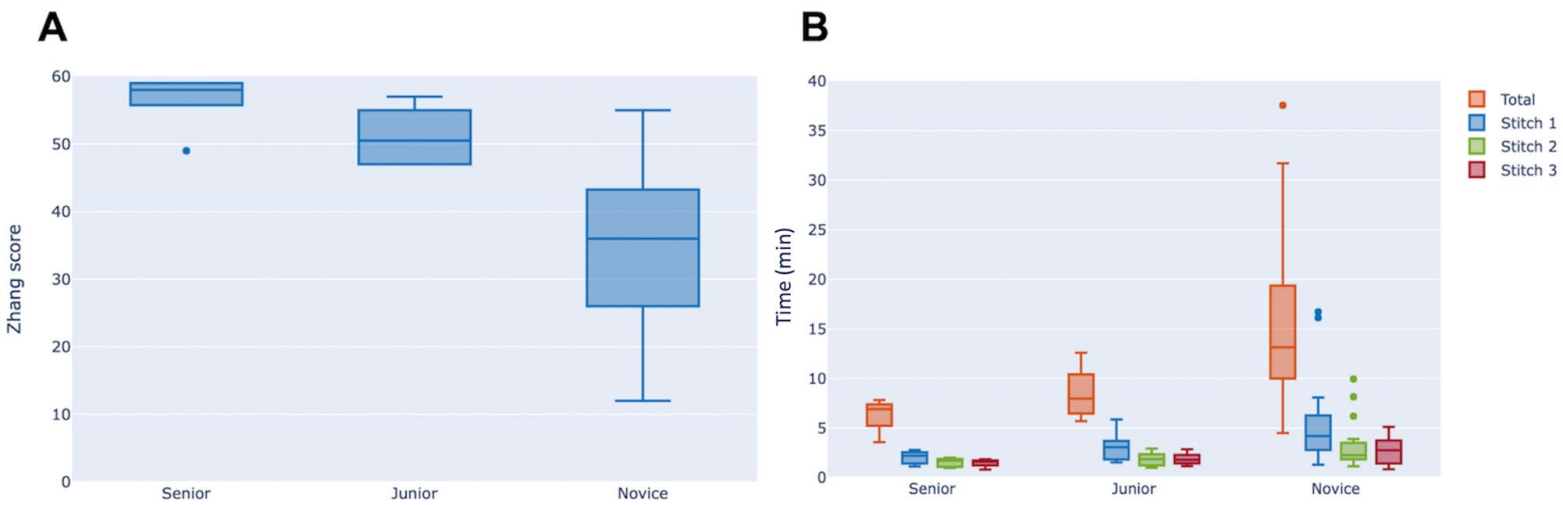
Box-and-whisker plot representing stitch quality assessment score (Zhang score) (**A**) and time analysis (**B**) for the three groups of the study (medians and interquartile ranges). (**A**) Zhang score significantly increased in more experienced groups (novice group < junior group < senior group; p < 0.001). (**B**) Total time with penalties to perform three corneal stitches (orange), time taken to perform the first (blue) and second (green) stitch significantly decreased in more experienced groups (p = 0.002, p = 0.013 and p = 0.032, respectively). Time to perform the third stitch (red) did not differ between groups. Data are represented as medians and interquartile ranges.

Zhang score for suturing performance increased significantly with participants’ expertise level (36.0 [28.0–43.0] for NG, 50.5 [47.0–54.5] for JG and 58.0 [58.0–59.0] for SG; p < 0.001) (Table 1 and Fig. 1A). RULA score for ergonomics assessment did not differ between groups (Table 1).

### Time analysis

Total time to achieve the wound closure decreased significantly with the participants’ level of expertise (13.1 [10.6–18.9] min for NG, 7.97 [6.75–9.84] min for JG and 6.90 [5.77–7.23] min for SG; p = 0.00185). This result remained true without time penalties (p = 0.0175).

Time to achieve a single stitch decreased during the session in all three groups. This yields a learning curve where the time difference from one stitch to another is, as expected, less significant for an SG participant than an NG participant. Only the NG participants required more time to perform their third stitch with respect to the second stitch (Table 1 and Fig. 1B).

### Zone analysis

**Figure 2:**
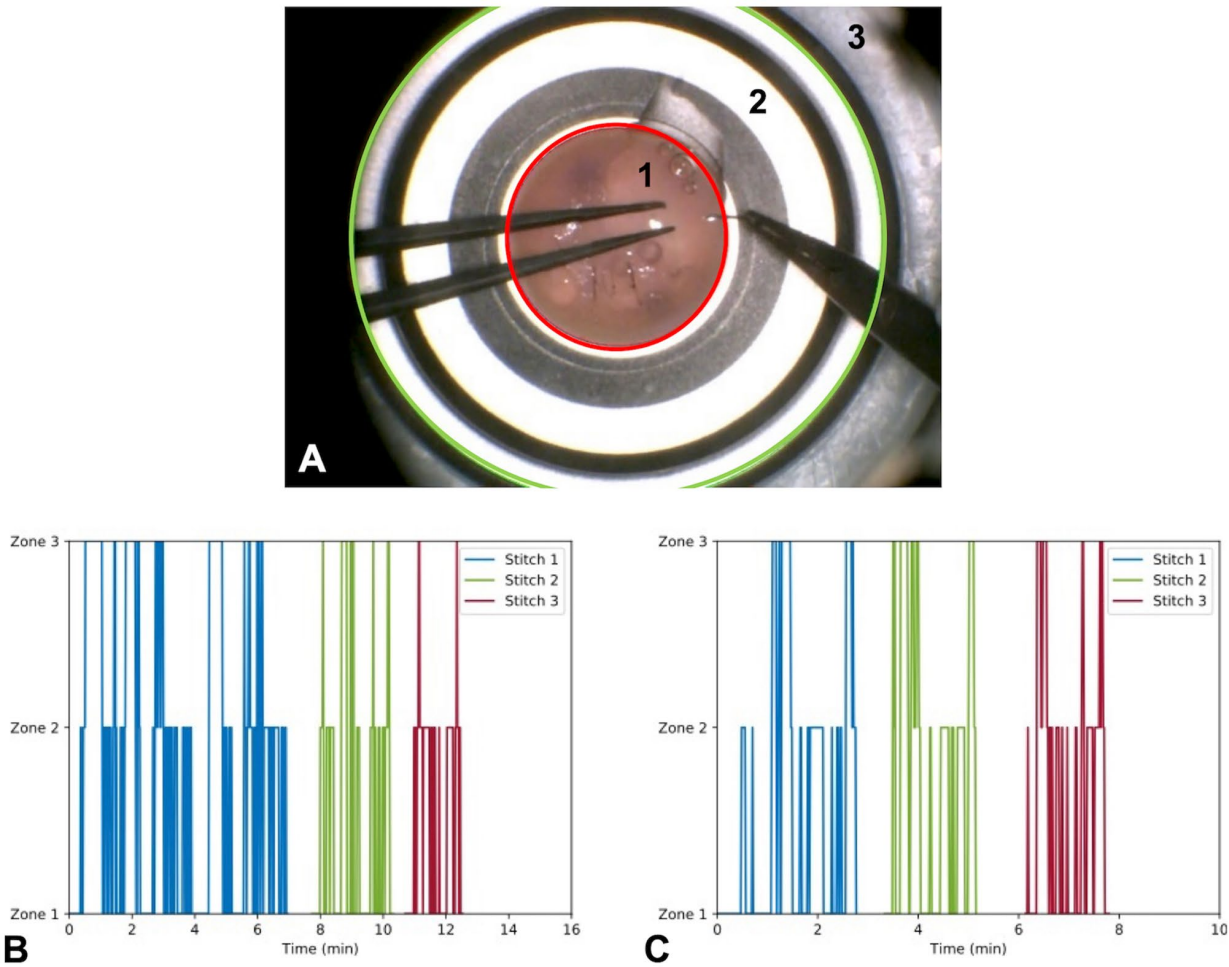
Zone analysis. (**A**) The microscope field of view was divided into three zones by drawing two separate circles: zone 1 corresponds to the surface of the cornea (red circle), zone 2 to the surface of the mounting base (green circle) and zone 3 to the outside of the mounting base. The position of surgical instruments within these zones was reported over time. (**B**,**C**) Plots representing the transition of the surgical tools within three different zones over time for a representative novice (**B**) and senior (**C**) participant.

Although comparisons of the percentages of time spent in each zone were not significantly different between groups, SG participants seemed to concentrate their performance time within zone 1 (69.2% of the total time on average). In contrast, JG and NG participants spent only 54.7% and 61.0%, respectively, of the total time in this zone (Table 1). SG participants seemed to minimize their back-and-forth movement between zone 1 and 2, when compared to NG participants (Fig. 2B,C). Time spent outside in zone 3 by SG participants correlated with the achievement of two specific tasks: pulling the thread after passing the needle through the cornea and changing of instruments before cutting the thread. NG participants, conversely, needed several passages in zone 3 to reposition the needle, grasp it with the needle holder or to shift the thread out of the field of view.

### Geometry analysis

**Figure 3:**
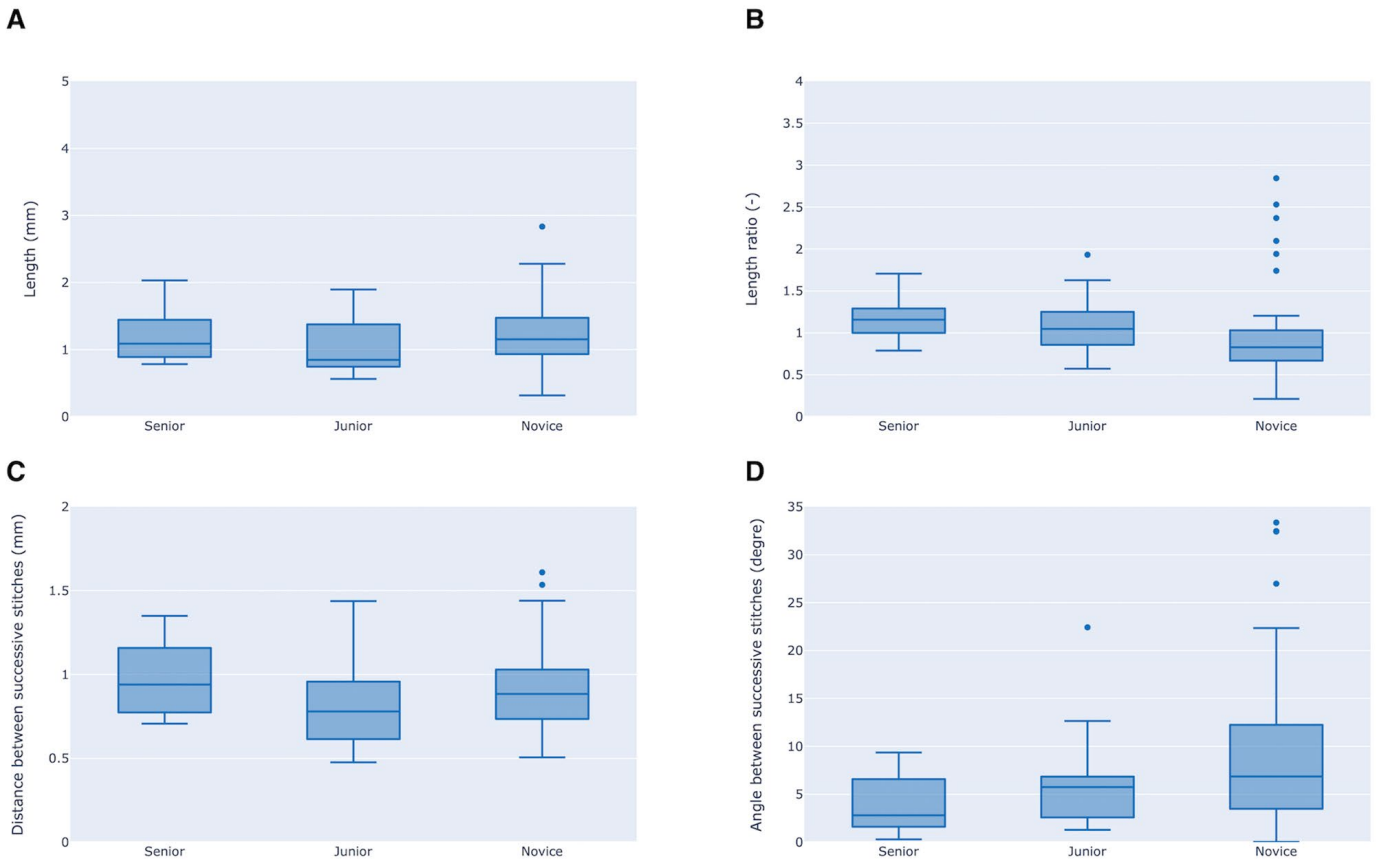
Box-and-whisker representing objective geometric assessments of the corneal stitches for three groups of surgeons. The length of stitches (**A**), the length ratio with respect to the first stitch (**B**), the distance between two successive stitches (**C**) and the angle between two successive stitches (**D**) are depicted. Length ratio with respect to the first stitch significantly increased in more experienced groups (novice group < junior group < expert group; p = 0.011). Data are represented as medians and interquartile ranges.

Outcomes of the four geometric criteria were compared between groups (Table 1). Length of the stitches, distance and angle between two stitches did not differ between groups (Fig. 3A,C,D). The length ratio of stitch 2 or 3 with respect to stitch 1 was significantly different between groups (Fig. 3B). With a length ratio close to 1, participants from the JG performed more regular stitches compared to the SG, in which length ratio increased over the course of the session (ratio of 1.05 [0.87–1.24] vs*.* 1.16 [1.03–1.28]; p = 0.0114). Stitch length decreased over the course of the session in the NG (ratio of 0.83 [0.67–1.02]).

### Confounding factors and survey

The prevalence of videogame and musical instrument playing, and the anxiety assessment scores were similar across groups (Supplementary Table 1). Subjective comfort with corneal suturing during sessions was significantly lower in the NG compared to the rest of the participants (5.0 ± 1.3 vs. 7.8 ± 2.4; p = 0.001), while participants from the JG had a significantly higher score than the other participants (8.0 ± 1.9 vs. 5.8 ± 2.3; p = 0.026). The NG subjective progression score was significantly higher than the other groups (3.3 ± 0.8 vs. 2.6 ± 0.8; p = 0.025), while the SG reported significantly less progression and utility scores compared to the other groups (2.2 ± 0.8 vs. 3.1 ± 0.7; p = 0.013 and 2.2 ± 0.8 vs. 3.2 ± 0.8; p = 0.01 for progression and utility scores, respectively).

## Discussion and conclusion

In the study discussed herein, three groups of ophthalmologists with differing experience in corneal suturing were prospectively compared during a three-stitch corneal wound closure task. The quality and geometry of the stitches, instrument handling, body ergonomics and time to perform the procedure were assessed.

The Zhang score efficiently differentiated between stitch quality between groups. Current assessment tools for corneal stitches quality remain underdeveloped and sometimes fail to differentiate between experienced and non-experienced surgeons^3,8^. The Zhang score enables an exhaustive assessment of corneal wound closure quality by precisely rating the completion of each consecutive stitch, fluency of movements, time required, geometry of the stitches, and efficacy of the wound closure^9^. Such multiple-parameters assessment enables a closer representation of “real-life” suturing tasks. From the 15 points initially proposed by Zhang et al*.*, three were removed: the “preoperative preparation”, “postoperative clean-up” and “knot rotation”. Although the first two were considered not relevant for this study, the “knot rotation” of one stitch might contribute to the ultimate effectiveness of the wound closure and should be evaluated in future assessments (Supplementary Fig. 1).

Musculoskeletal disorders have been reported in ophthalmic surgeons, indicating that ergonomic practices are of major importance during surgical training^10^. In this study, ergonomics was assessed using the RULA score, which provides an evaluation of the surgeon’s posture (head, trunk posture, and upper limbs, up to the wrist) while seated^11^. This score did not differ between groups. However, it is questionable if RULA is applicable to microsurgical procedures, as it does not allow for a precise evaluation of small movements. The evaluation of the head position at three different angles and hand movements considering only wrist position may be too simplified. Moreover, the assessment of flexion and extension angles of subtle body movements (for example, in the forearms or wrist), might have proved challenging for assessors, leading to overly subjective evaluations.

Ratings of Zhang and RULA scores was time consuming (multiple-step analysis with multiple scoring). Automation of these assessments would allow rapid, objective and reproducible analysis of the suturing task. Suturing skills assessments could rely on predetermined parameters to be assessed by artificial intelligence. The ergonomics assessment should be carried out by a 3D-recording of hand and finger motions. The Imperial College Surgical Assessment Device (ICSAD) allows 3-dimensional recording of index finger movements during ophthalmic surgery. However, the device is described as cumbersome, difficult to install and expensive^6^. Further studies on similar, but more affordable, alternative systems should be undertaken. An automated assessment, with directly available results, would motivate trainees to perform procedures independently. Moreover, novices’ comfort and confidence could be enhanced by the possibility of practicing in the absence of senior supervision.

Total time to perform the procedure was significantly lower in senior surgeons compared to juniors and novices. Such data is relevant in current practice, as lowering the duration of one surgical procedure increases its quality^12^. Regarding the time taken to complete each of the three stitches, no difference was observed between the JG and SG, while the NG participants required more time to perform the third stitch with respect to the second stitch. This result might reflect the effects of fatigue when approaching the end of the procedure, or an increased degree of stress rendering the procedure difficult to finish. The lesser experience in this group most likely explains this result. Subjective scoring by participants showed significantly lower initial comfort during the suturing sessions in the NG. Intermediate time did not differ between groups. This finding should be considered in parallel with the analysis of the participants’ handling of surgical tools.

The analysis of instrument handling revealed that the SG participants mostly concentrated their performance time in zone 1, while the JG and NG spent less time in this zone. The SG participants seemed to minimize their back-and-forth movement between zone 1 and 2 when compared to the NG participants. These data show that more experienced surgeons probably focus their movements on the target zone and save time outside of the operation field. In the literature, there are reports of real-time visual tracking of surgical instruments using specific algorithms, instrument modelling or motion analysis software, which could be a way to assess instrument handling more accurately^13,14^. When observing room cameras, time spent out of the operation field could be analyzed. The instruments handling analysis may help to differentiate economy of movement and efficiency of the procedure between groups. However, it should be combined with the recording of body movements, using cameras placed in the operation room (OR). Body movements could be assessed by counting the number of movements of significant amplitude in a predefined direction. Automated counting could be performed using a computer program, as previously reported, applying selective labelling of tools or body parts of interest^15^. There are also entirely different methods for body movement assessment, such as applying electromyogram (EMG) sensors to the limb and torso for evaluating different postures of the body during surgery^16^.

Analysis of stitches geometry showed that the JG participants performed more regular stitches, in terms of length, compared to the SG, whose stitch length increased over the course of the session. If one considers that a suitable corneal stitch should be composed of 2 mm long stitches, all participants completed undersized stitches, including senior participants. These results are surprising, yet the training conditions might have disturbed experienced surgeons who are used to OR conditions. Several factors could have contributed to this, such as the absence of a patient forehead due to the use of an artificial anterior chamber. Nevertheless, considering that a 1 mm distance remains satisfactory, the SG participants respected this interval best and performed the most parallel stitches compared to the other participants. Although these results did not clearly between novices or juniors and senior surgeons, an automated and fast analysis of stitches characteristics may be a promising method for the follow up of residents’ performances. Longer procedures and closer-to-OR situations should help demonstrate this.

Subjective scales allowed to clarify trainees’ feelings and impressions of the procedure. Surprisingly, initial comfort in corneal suturing was not highest in the SG. This may, again, be due to the different environment compared to usual OR conditions. Anxiety scores were similar between groups. Reproduction of exact OR conditions (sounds, lighting, and timing of anesthetic drugs effect), may result in differences between the groups, with novices likely being more anxious in this situation. Previous experience with video games and musical instruments did not correlate with better microsurgical skills, which is consistent with previous reports in microsurgery^17^.

In this validity study for a multiple metrics assessment method, the most differentiating and valuable metrics for corneal suturing skills assessment were the following: (1) a twelve-point assessment scale of corneal wound closure quality (modified Zhang score); (2) detailed time record (time to perform the entire surgical procedure and time needed for each step); and (3) a semi-automated assessment of tool handling using video records from the microscope. This latter assessment might be combined with body-and-hand motion analysis for more accuracy, as described above. Other metric assessment methods used in this study (i.e., ergonomics assessed by RULA and stitches geometry analysis) lacked enough power for significant value. Ergonomics assessment should rely on a more accurate analysis of head and hand motion. Corneal stitches quality assessment should be performed by an automated, fast and easy-to-manipulate system, improving reproducibility and saving time for the assessors. Additionally, the general conditions of the training session should approach real OR conditions. Our results could be confirmed on longer and more complex suturing procedures (transfixing keratoplasty or strabismus surgery) with adequate models and conditions. While human corneal grafts closely reproduce the characteristics of living corneas, they represent a limited resource. This should promote the design of realistic, artificial cornea. Finally, in our study, several weeks had lapsed between the three sessions of corneal suturing, which might have prevented the possibility to observe session-by-session improvement, notably in the NG. Reducing the time between sessions would allow to assess and compare this improvement between groups.

The present validity study reports a multiple metrics assessment method for reliable evaluation of corneal suturing skills. Several items from this method efficiently distinguished between three groups of surgeons with different experience performing a corneal wound closure task, including a wound closure quality scale, detailed time record and tool handling analysis. This paves the way for future efficacy studies to confirm the method’s benefit on corneal suturing skills training. Finally, future improvements of these methods might include the use of automated systems, longer suturing procedures, and close-to-OR environments to increase the accuracy and reproducibility of the evaluation.

## Materials and methods

This monocentric, prospective study was conducted on the premises of GEPROMED within Strasbourg University Hospital. Ethics approval was obtained by the Ethics Committee of Strasbourg University Hospital. All Methods were performed in accordance with the relevant guidelines and regulations. All training sessions were performed in a dedicated surgical room. Participants were enrolled in the study between November 6, 2019 and November 21, 2019. This study was considered a validity study for the characterization of skills assessment methods in corneal suturing^2^.

### Materials

**Figure 4:**
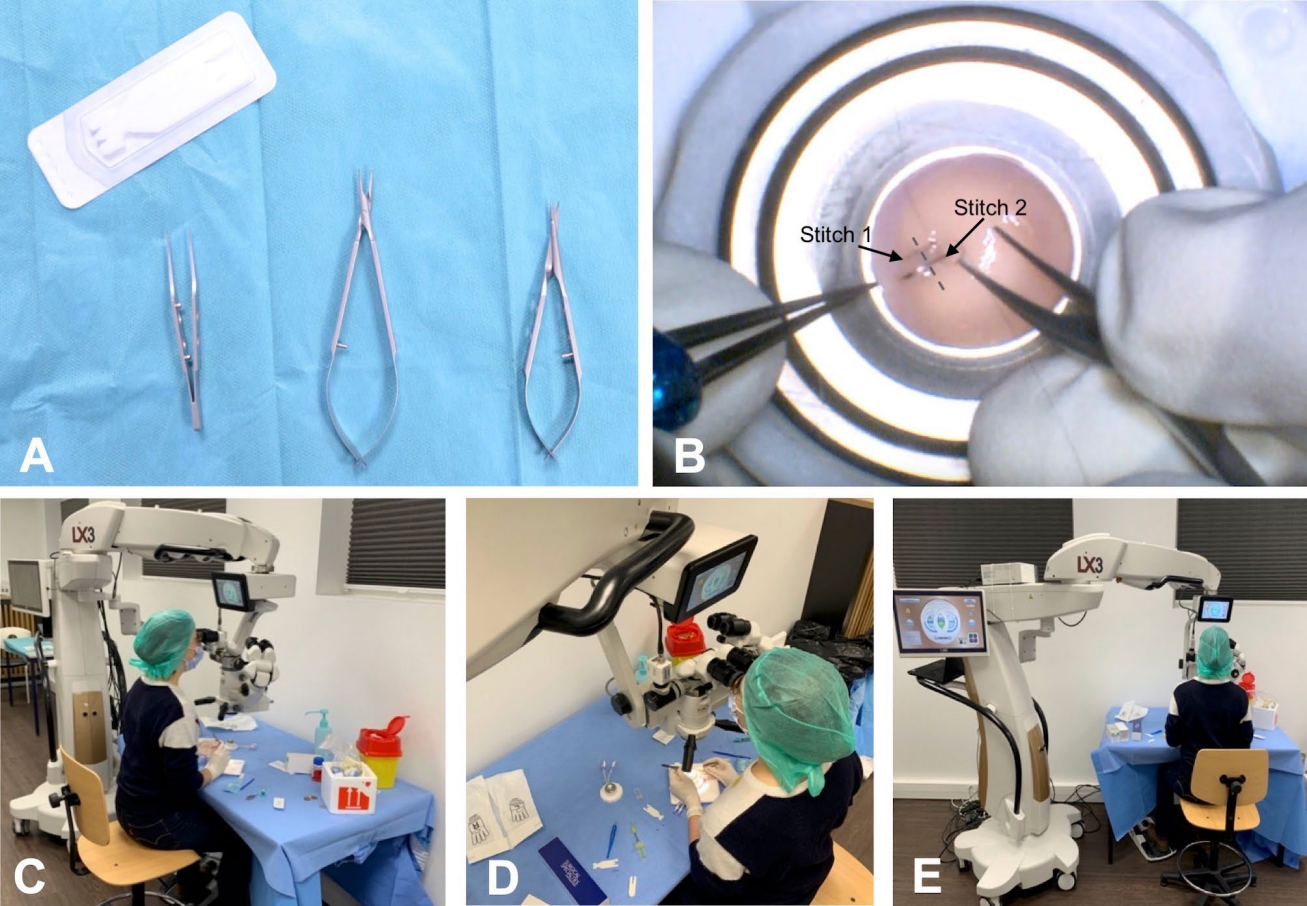
Surgical training instruments and recording. (**A**) Instruments used during training sessions: 10-0 Ethilon Nylon suture threads (Ethicon, US, LLC, top left). At the bottom of the image, from left to right: Bonn forceps (left), Barraquer needle holder (middle) and Vannas scissors (right). (**B**) Microscope camera view showing one paracentral corneal wound (grey dotted line) being closed by separated stitches. The first stitch is completed (stitch 1, black arrow), while the second stitch is in progress with the needle already introduced into the cornea (stitch 2, black arrow). (**C**) Room camera showing the participant’s arm, forearm and trunk position. (**D**) Room camera showing the participant’s hand and wrist movements. (**E**) Room camera showing participant’s head, back and leg alignment.

Human corneal transplants considered unsuitable for surgical use (with large scleral rims and low endothelial cell densities) were provided by the EFS Bourgogne-Franche-Comté Cornea Bank (Besançon, France). The transplants were placed in an artificial anterior chamber (Moria SA, Antony, France). The chamber was filled with a viscoelastic substance (Viscoat, Alcon, TX, United States) to achieve an intraocular pressure ranging between 15- and 20 mmHg, estimated by digital palpation. Procedures were performed under an operating microscope (Luxor LX3, Alcon, TX) using 10-0 Ethilon Nylon suture threads (Ethicon, US, LLC) and a set of sterilizable microsurgical suture instruments (Fig. 4A). The microscope was installed vertically above the camera, reproducing standard ocular surgery conditions. Each session was video recorded by the microscope camera (Fig. 4B). The training room was video recorded by three ceiling-mounted cameras at different angles for the ergonomics assessment (Fig. 4C–E). Video recordings of each participant were randomly allocated a serial number to ensure blindness of the review.

### Participants

Volunteer ophthalmologists of all levels of experience in microsurgery were enrolled after providing written informed consent. Prior to the start of the study, participants were allocated to three different groups, according to their level of experience in corneal surgery. Participants who had previously carried out more than 100 corneal stitches were assigned to the “Senior Group” (SG), those who had performed 10 to 100 corneal stitches in the “Junior Group” (JG) and those who had performed less than 10 in the “Novice Group” (NG). All completed three training sessions of corneal wound suturing.

### Surgical training

For each session, participants were asked to repair a 4-mm linear penetrating wound with three stitches. One paracentral corneal wound was created manually on the transplant using a 30° surgical blade. Three stitch loops had to be done for each stitch. During their first session, novice surgeons were taught about corneal suturing methods and allowed to ask for further help during the procedure. Participants were able to finish the procedure at their own pace and could ask to end the procedure before finishing if they felt that the task was too difficult to achieve.

### Assessment of the surgical training

The number of stitches successfully performed out of the three required was reported.

Assessments of suturing and ergonomics were carried out on the recorded videos by two blinded experienced ophthalmologists. Scoring of the suturing performance was carried out using a previously reported assessment scale (Zhang score), divided in 12 parts, each of which was rated on a 5-point Linkert scale, leading to a 60-point score (Supplementary Fig. 1)^9^. The ergonomics assessment was realized using a score validated for the investigation of work-related upper limb disorders, the “Rapid Upper Limb Assessment” (RULA) score^11^.

Total time taken to perform stitches was measured (from the first entry of the needle in the cornea to the last thread cut). Participants who could not finish the work were attributed a penalty of 300 s per uncompleted stitch to avoid any confusion between a premature termination or a fast achievement of the procedure. The time to realize each stitch (from the entry of the needle in the cornea to the thread cut) was also calculated, as was the intermediate time, representing the mean time elapsed between the performance of two stitches.

A semi-automated assessment of the stitches’ final geometry was performed by computing the length of each stitch and the angle and distance between two successive stitches using a picture of the cornea acquired at the end of the session. Surgical tool handling while working with the microscope was graded by considering the tools’ position relative to the center of the cornea over time. Three zones were defined in the microscope working area. Zone 1 represented the surface of the cornea, zone 2 the surface of the mounting base, excluding the surface of the cornea, and zone 3 the outside of the mounting base (Fig. 2A). Recorded videos were sampled at a frequency of 1 Hz. Each resulting image was attributed a zone number, according to the position of tools in the field of view. When none of the tools were visible in the field of view, zone 3 was allocated.

### Confounding factors

As all participants were allowed to continue their regular surgical activities for the duration of the study, they were asked to report the number of corneal stitches they performed between each session. Video game or musical instrument playing was also screened. Participants took both the STAI-YA and STAI-YB for the assessment of state and trait anxiety, respectively^18,19^. They also completed a survey, gathering their subjective comfort in corneal suturing during sessions (out of 12 points), impression of progress during the sessions (4 points) and opinion of the future utility of these sessions in their surgical practice (4 points) (Supplementary Table 2).

### Statistical analysis

Statistical analysis was performed using the *scipy.stats* module under Python (version 3.7.4). Statistical comparisons between the three groups were performed by Kruskal–Wallis test and by the Mann–Whitney test when comparing two groups. Results were considered statistically significant if p-value < 0.05.

### Ethics approval and consent to participate

Ethics approval was obtained by the Ethics Committee of Strasbourg University Hospital. Participants were enrolled after providing written informed consent.

## Supplementary Information

Supplementary Figure 1.

Supplementary Table 1.

Supplementary Table 2.

## Data Availability

The datasets used and analyzed during the study reported herein are available from the corresponding author on reasonable request.
