# Supplementary material for: Multiple metrics assessment method for a reliable evaluation of corneal suturing skills
Source: Sci Rep. 2023 Feb 20;13:2920. doi: 10.1038/s41598-023-29555-3 (PMC9941077; doi:10.1038/s41598-023-29555-3)
Supplement: Supplementary file 1 — Supplementary Figure 1. [file 41598_2023_29555_MOESM1_ESM.pdf]

	1	2	3	4	5	Score
**Microscope use**	Out of center and focus constantly	→	→	→	Centered and focused constantly	
**Instrument handling**	Constant tentative and awkward moves with instruments	→	→	→	Fluid moves with instruments	
**Hands coordination**	Severe hands tremor and constant instruments collision	→	→	→	Steady with perfect hands coordination	
**Suturing**	Great difficulty and slow fashion to make sutures	→	→	→	Smooth and perfect suturing	
**Suturing order**	Suture the rupture randomly	→	→	→	Selectively suture the rupture	
**Stitches interval**	Awfully uneven	→	→	→	Perfectly even (2 mm)	
**Stitches width**	Awfully uneven	→	→	→	Perfectly even (2 mm)	
**Stitches depth**	Awfully uneven	→	→	→	Perfectly even (2/3 of corneal thickness)	
**Knotting**	Great difficulty and slow fashion to place knots	→	→	→	Knots placed perfectly with no breaking sutures	
**Knots tightness**	Awfully uneven tightness: too tight or loose sutures	→	→	→	Perfectly even tightness. No astigmatism induced	
**Wound closure and anterior chamber formation**	No wound closure and no anterior chamber formation	→	→	→	Neat and watertight wound closure. Perfect anterior chamber formation	
**Overall performance**	Unable to finish the operation independently	→	→	→	Confident and fluid, finish the operation within 5mins	
					**Total score (/60 points)**	

Supplementary Figure 1. Twelve-items modified Zhang score for the assessment of a 3-millimeters long corneal wound closure. Each item is rated on a 5-point Linkert scale, one being the worst and 5 the best rate, leading to a 60-points score (adapted from Zhang et al.[9]).
