# Supplementary material for: Multiple metrics assessment method for a reliable evaluation of corneal suturing skills
Source: Sci Rep. 2023 Feb 20;13:2920. doi: 10.1038/s41598-023-29555-3 (PMC9941077; doi:10.1038/s41598-023-29555-3)
Supplement: Supplementary file 2 — Supplementary Table 1. [file 41598_2023_29555_MOESM2_ESM.docx]

| Group | Number of participants | Anxiety scores Median (interquartile range) | | Number of participants with specific background | |
| --- | --- | --- | --- | --- | --- |
|  |  | **Anxiety state (STAI-YA)** | **Anxiety trait (STAI-YB)** | **Video games** | **Musical instruments** |
| Senior Group | 5 | 38 (30-43) | 43 (34 - 46) | 2(40%) | 1(20%) |
| Junior Group | 8 | 38 (36-42) | 45 (43-49) | 5(63%) | 4(50%) |
| Novice Group | 33 | 43 (35-49) | 42 (41-47) | 5(45%) | 4(36%) |

STAI: State Trait Inventory Anxiety

**Supplementary Table 1.** Results of anxiety score (state and trait) and previous history of video games or musical instrwument playing concerning the three studied groups. The level of anxiety is considered as very weak for a STAI-Y score <35, weak from 36 to 45, average from 46 to 55, high from 56 to 65, and very high for a score >65.
