# Supplementary material for: Multiple metrics assessment method for a reliable evaluation of corneal suturing skills
Source: Sci Rep. 2023 Feb 20;13:2920. doi: 10.1038/s41598-023-29555-3 (PMC9941077; doi:10.1038/s41598-023-29555-3)
Supplement: Supplementary file 3 — Supplementary Table 2. [file 41598_2023_29555_MOESM3_ESM.docx]

| Question | Please indicate the most appropriate answer | | | |
| --- | --- | --- | --- | --- |
|  | **No** | **Rather no** | **Rather yes** | **Yes** |
| 1. I felt comfortable with stitches achievement starting the suturing sessions |  |  |  |  |
| 2. I felt immediately fast enough for stitches achievement during sessions |  |  |  |  |
| 3. I felt the quality of my stitches was immediately satisfying during sessions |  |  |  |  |
| 4. I think that my suturing skills increased throughout the suturing sessions |  |  |  |  |
| 5. I think that the suturing sessions will be useful for my future surgical practice |  |  |  |  |

**Supplementary Table 2.** Survey to be completed by the study participants, gathering subjective comfort in corneal suturing (items 1, 2 and 3), impression of progress during sessions (item 4) and opinion of the future utility of these sessions in daily surgical practice (item 5). For each line, the selection of “No” scores 1 point, “Rather no” 2 points, “Rather yes” 3 points and “Yes” 4 points, so that each line is assessed on a 4-points score. Items 1, 2 and 3 are secondarily gathered on a 12-points score for the study.
